# VALIDATION OF SCREENING SYSTEMS IN PEDIATRIC EMERGENCY

**DOI:** 10.1590/1984-0462/;2018;36;4;00018

**Published:** 2018

**Authors:** Emílio Carlos Elias Baracat

**Affiliations:** a Universidade Estadual de Campinas, Campinas, SP, Brasil.

The main objective of screening in pediatric emergency services is to determine and classify care priority, avoiding the worsening of clinical conditions of critically ill patients. In a rapid approach, signs and symptoms are identified and a five-level stratification of urgency categories is made, helping define priorities and waiting time.^1^ The most commonly used international screening systems are The Manchester Triage System (MTS), Emergency Severity Index (ESI) version 4, The Pediatric Canadian Triage and Acuity Scale (PaedCTAS), and Australian Triage Scale (ATS), the first three with specific chapters addressing the pediatric population.^2^ ^,^ ^3^ ^,^ ^4^

The validation of screening systems is determined by reliability (inter-observer and intra-observer agreement) based on the Kappa coefficient, and by the instrument’s ability to predict “actual urgency” (internal validation). As “actual urgency” is hard to define, studies use indicators of severity such as hospitalization, admission to the Intensive Care Unit (ICU), resources used for the diagnosis and length of stay in the emergency room.^4^ ^,^ ^5^ ^,^ ^6^ In addition, a screening system can be validated based on sensitivity/specificity, that is, the capability to identify patients in high- or low-urgency clinical conditions. Finally, the external validation refers to the applicability of the instrument to different environments and scenarios.

The literature on validation and reliability of screening systems shows moderate/good results when applying ESI and PaedCTAS to children.^4^ ^,^ ^7^ In a recent study that included three European centers, MTS validation showed sensitivity of 0.65-0.83 and specificity of 0.83-0.89, with worse performance when applied to young children. A marked variation in performance across different research centers was also shown, indicating an ongoing need for improvement.^8^

In this issue of *Revista Paulista de Pediatria*, Magalhães Barbosa and colleagues present a validation/reliability study of a system known as CLARIPED, which stands for Risk Classification in Pediatric Emergencies, and was proposed in a recent publication.^9^ Following the methodologies used in other studies on this subject, the authors used two validation methods: risk classification determined by the system, based on clinical outcomes, and comparison with a reference standard based on MTS. The findings showed good agreement and high sensitivity and specificity to categorize very urgent levels. As to reliability, Kappa values were similar to those of the three most commonly used screening systems in this population.

Multicenter studies on CLARIPED applied to different demographic realities, pediatric emergency services of varying complexity and with large cohorts of patients with a variety of diagnoses and disease degrees are considered the most promising ones on a subject with such high priority and this current.
